# Artificial Intelligence in Oral Diagnosis: Detecting Coated Tongue with Convolutional Neural Networks

**DOI:** 10.3390/diagnostics15081024

**Published:** 2025-04-17

**Authors:** Sümeyye Coşgun Baybars, Merve Hacer Talu, Çağla Danacı, Seda Arslan Tuncer

**Affiliations:** 1 Department of Oral and Maxillofacial Radiology, Faculty of Dentistry, Firat University, Elazığ 23119, Turkey; mhduran@firat.edu.tr; 2 Department of Software Engineering, Faculty of Engineering, Firat University, Elazığ 23119, Turkey; cdanaci@firat.edu.tr (Ç.D.); satuncer@firat.edu.tr (S.A.T.)

**Keywords:** coated tongue, convolutional neural network, deep learning, machine learning, oral diagnosis, support vector machine

## Abstract

**Background/Objectives:** Coated tongue is a common oral condition with notable clinical relevance, often overlooked due to its asymptomatic nature. Its presence may reflect poor oral hygiene and can serve as an early indicator of underlying systemic diseases. This study aimed to develop a robust diagnostic model utilizing convolutional neural networks and machine learning classifiers to improve the detection of coated tongue lesions. **Methods:** A total of 200 tongue images (100 coated and 100 healthy) were analyzed. Images were acquired using a DSLR camera (Nikon D5500 with Sigma Macro 105 mm lens, Nikon, Tokyo, Japan) under standardized daylight conditions. Following preprocessing, feature vectors were extracted using CNN architectures (VGG16, VGG19, ResNet, MobileNet, and NasNet) and classified using Support Vector Machine (SVM), K-Nearest Neighbors (KNN), and Multi-Layer Perceptron (MLP) classifiers. Performance metrics included sensitivity, specificity, accuracy, and F1 score. **Results:** The SVM + VGG19 hybrid model achieved the best performance among all tested configurations, with a sensitivity of 82.6%, specificity of 88.23%, accuracy of 85%, and an F1 score of 86.36%. **Conclusions:** The SVM + VGG19 model demonstrated high accuracy and reliability in diagnosing coated tongue lesions, highlighting its potential as an effective clinical decision support tool. Future research with larger datasets may further enhance model robustness and applicability in diverse populations.

## 1. Introduction

Coated tongue is characterized by a painless, white or whitish-yellow layer on the dorsum of the tongue. This coating consists of desquamated epithelial cells, bacteria, blood metabolites, secretions from the nasopharynx and gingiva, as well as saliva. Coated tongue commonly appears during febrile conditions such as scarlet fever, primary herpetic gingivostomatitis, multiple aphthous ulcers, and bullous diseases—clinical scenarios that are frequently associated with painful oral lesions. In elderly individuals, the coating tends to be thicker and may present with a more atypical coloration compared to younger populations. Additionally, coated tongue has been reported as a potential risk factor for aspiration pneumonia in individuals aged 65 years and older [1,2]. Furthermore, individuals with gastrointestinal or hepatic disorders have been observed to exhibit a thicker tongue coating than healthy individuals [3]. Differential diagnoses should include conditions such as hairy tongue and oral candidiasis; however, histopathological examination is not required in most cases [1]. Epidemiological studies conducted in different populations indicate that the prevalence and presentation of tongue lesions vary across communities [4,5]. Therefore, understanding and identifying the prevalence of tongue lesions specific to each population is of great importance for the development of regionally tailored public health policies. Patients often visit dentists primarily due to tooth pain. However, since dentists tend to focus on pain management and treatment of the patient’s main complaints, comprehensive examinations of the oral mucosa and tongue are often overlooked. As coated tongue and other tongue lesions are usually asymptomatic, they are frequently neglected or left untreated as potential underlying causes of symptoms such as halitosis, burning mouth sensation, and taste disorders. Recent advancements in artificial intelligence (AI) have led to a profound transformation in the healthcare field, enabling the development of innovative approaches for the diagnosis, management, and prevention of various diseases [6,7,8]. Subfields of AI, particularly machine learning and deep learning, make it possible to develop highly accurate diagnostic tools by identifying complex patterns and relationships within large medical datasets [9,10]. In particular, convolutional neural network (CNN) models based on deep learning have become some of the most widely used techniques in medical imaging, supporting advanced applications such as organ segmentation, detection, classification, and disease diagnosis [11,12]. In the field of dentistry, these models have been applied to the detection of caries, lesions, and cysts, as well as in treatment planning [7,13,14]. Additionally, systemic conditions with oral manifestations and various oral diseases have been analyzed using artificial neural networks trained on intraoral photographs, demonstrating the diagnostic and clinical decision-support potential of AI in such contexts [8,15]. In conventional tongue examinations, external factors such as lighting conditions, the ambient environment, and even seasonal variability may lead to inconsistencies in diagnostic outcomes. CNNs, however, learn features directly from data, thereby minimizing the impact of such external variations and providing a more consistent diagnostic process. The fundamental goal of these networks is to optimize the weight parameters across layers to achieve high levels of accuracy and generalizability [16,17].

This study aims to develop an innovative decision support system to assist clinicians in the diagnosis of coated tongue lesions. By adopting a hybrid approach that integrates deep learning and machine learning techniques, the study seeks to enhance diagnostic accuracy, reduce subjective assessments, and offer a reliable tool for clinical applications.

The contributions of this study to the literature are summarized below in bullet points.

- It aims to provide an AI-assisted approach for the diagnosis of coated tongue, a commonly overlooked oral condition, thereby promoting early detection and awareness.
- A new data set has been added to the literature and shared.
- A highly accurate diagnostic model has been developed by integrating deep learning-based CNN architectures with traditional machine learning classifiers.
- The superior performance of the VGG19 + SVM combination offers valuable insights into effective model configurations for similar problems in the literature.
- It contributes to the development of reliable, automated diagnostic systems that can be used as supportive tools in clinical applications.
- By introducing a standardized approach to intraoral imaging and evaluation, it has the potential to reduce the impact of external factors (e.g., lighting, seasonal variations) on diagnostic consistency.
- The early detection of coated tongue, which has been linked to serious conditions such as aspiration pneumonia in individuals aged 65 and over, is emphasized as a public health priority.
- It provides a new perspective on the diagnostic evaluation of coated tongue in relation to gastrointestinal, hepatic, and systemic diseases.

## 2. Materials & Methods

### 2.1. Patient Selection and Image Acquisition

**Figure 1:**
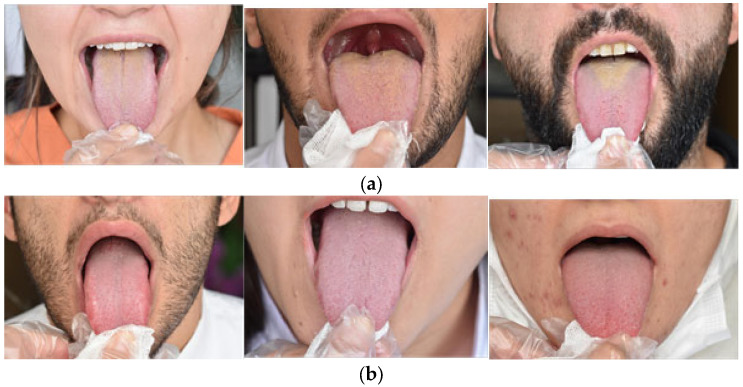
Coated tongue images (**a**), healthy tongue images (**b**).

This study utilized tongue images obtained during routine examinations of patients presenting with various complaints to the Department of Oral and Maxillofacial Radiology at the Faculty of Dentistry, Fırat University. The dataset consisted of 200 tongue images, evenly divided between 100 coated tongues and 100 healthy tongues, with stabilization achieved using gauze. All images were captured under standardized natural daylight conditions using a professional single-lens reflex camera (Nikon D5500 with Sigma Macro 105 mm lens, Nikon, Tokyo, Japan). Image acquisition was performed by an oral and maxillofacial radiology research assistant (M.H.T.) and an oral and maxillofacial radiologist (S.C.B.). To ensure optimal image quality, any out-of-focus images were excluded. The final dataset images were saved in JPEG format, in RGB color mode, with a resolution of 6000 × 4000 pixels and an uncompressed 1:1 aspect ratio. Figure 1a provides examples of coated tongues, while Figure 1b illustrates healthy tongues.

### 2.2. Image Evaluation

**Figure 2:**
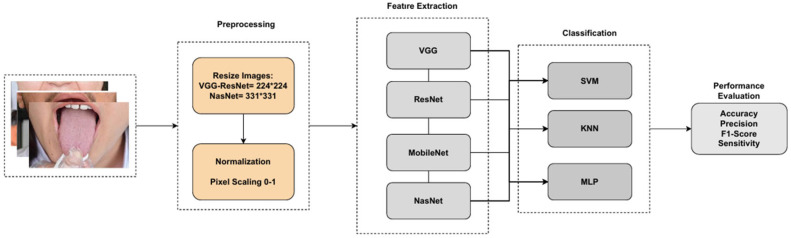
The proposed method.

The dataset used for the diagnosis of coated tongue consists of tongue photographs taken from individuals with healthy and coated tongues. These images were used for direct classification without segmentation. This approach prioritizes distinguishing between “coated” and “healthy” tongues, eliminating the need for segmentation and providing practical advantages. CNN models require input data of certain sizes according to their architectural designs. To meet these requirements, the first step of the study included standardizing the dataset by resizing all images. The images were set to 224 × 224 pixels for the VGG, ResNet, and MobileNet architectures and 331 × 331 pixels for NasNet. Additionally, the images were normalized by scaling the pixel values to a range between 0 and 1 using min-max normalization to speed up the learning process of the model and ensure more balanced weight updates. After the preprocessing steps, the CNN models (VGG16, VGG19, ResNet18, ResNet50, MobileNet, and NasNet) had weights trained on the ImageNet dataset and were applied for feature extraction with the transfer learning method. Feature extraction was performed in the last convolutional layer of each model, which showed the best discriminative features. In VGG models, a “flattening” layer is used, especially before the “fc7” fully connected layer. In ResNet models, features are obtained using the last global average pooling (GAP) layer. In MobileNet and NasNet models, features are extracted using deep discrete convolutional layers. After the feature extraction processes, a feature vector was created for each image and given as input to the proposed hybrid structure machine learning classifiers. Thus, the features required for the classification process were obtained by utilizing the high representation power of different CNN models. The flow of the proposed method is given in Figure 2. The features obtained from CNN models were classified using traditional machine learning methods. At this stage, Support Vector Machine (SVM), K-Nearest Neighbors (KNN), and Multilayer Perceptron (MLP) algorithms were included in the study.

RBF kernel was used while performing hyperparameter tuning for the SVM model. The C parameter, which determines the complexity of the model, was set to 1 and the gamma parameter was set to automatic. The number of neighbors for the KNN model was determined as k = 5 by performing k = (3,5,7) trials. The MLP model was designed in a structure with a single hidden layer, and 128 neurons and the Adam optimizer were used.

In order to increase the reliability of the study, separation of the training and test data was performed using the balanced separation method so that there was an equal number of examples from each class. The data set was divided into 80% training and 20% test. Thus, the ratio of classes in the data set was preserved and the model was not affected by the unbalanced data distribution. In addition, during the model evaluation, a five-fold cross-validation method was applied to ensure the reliability of the results and to increase the generalization of the model. Classifier performance was evaluated using accuracy, precision, recall, and F1 score measurements. By integrating CNN-derived features with various classifiers, the study achieved high accuracy rates while increasing model generalizability. This hybrid approach has proven to be critical in improving both the accuracy and robustness of the diagnostic model.

## 3. Results

An evaluation of the findings was conducted using sensitivity, accuracy, F1 score, and specificity metrics. The combined application of these metrics provided a comprehensive assessment of model performance, both in terms of overall accuracy and inter-class distinction. Analyzing these metrics collectively established a reliable foundation for identifying model deficiencies and determining the best-performing configuration. The formulas for the metrics used in the performance evaluation process are presented in Table 1.

- True Positive (TP): The number of images correctly identified as “coated” among the images of coated tongues.
- True Negative (TN): The number of images correctly identified as “healthy” among the images of healthy tongues.
- False Positive (FP): The number of images incorrectly identified as “healthy” among the images of coated tongues.
- False Negative (FN): The number of images incorrectly identified as “coated” among the images of healthy tongues.

The performance metrics obtained after the classification process are summarized in Table 2. This table was utilized to compare the different models and identify the one with the highest overall performance.

Upon analyzing Table 2, it is evident that multiple hybrid models achieved identical accuracy rates. In such cases, a comprehensive evaluation incorporating all performance metrics provided a more reliable basis for a comparative analysis. When all metrics were considered collectively, the SVM + VGG19 hybrid model demonstrated the highest overall performance. The calculated performance metrics for this model were as follows: 82.6% precision, 88.23% specificity, 85% accuracy, and an F1 score of 86.36%. These results highlight the model’s balanced approach to minimizing both false positives and false negatives, ultimately achieving a high level of classification performance.

In this study, the Support Vector Machine (SVM) method is recommended because, although similar accuracy rates were obtained with the Multi-Layer Perceptron (MLP), SVM offers several advantages. These include lower computational requirements, fewer hyperparameters to tune, and more stable performance, particularly on small to medium-sized datasets. Additionally, SVM provides more clearly defined decision boundaries, enhancing the interpretability of the model and reducing the risk of overfitting. Therefore, among models with comparable performance, SVM stood out as the most suitable and reliable choice.

The results indicate that the proposed method, utilizing the SVM + VGG19 hybrid model, reliably diagnosed coated tongue and effectively distinguished between coated and healthy tongues. The model’s high specificity reflected a low false positive rate for healthy individuals, thereby reducing the risk of misdiagnosis. Additionally, the elevated F1 score highlighted the model’s ability to achieve a well-balanced performance between precision and recall (sensitivity), underscoring its overall robustness in classification tasks.

**Figure 3:**
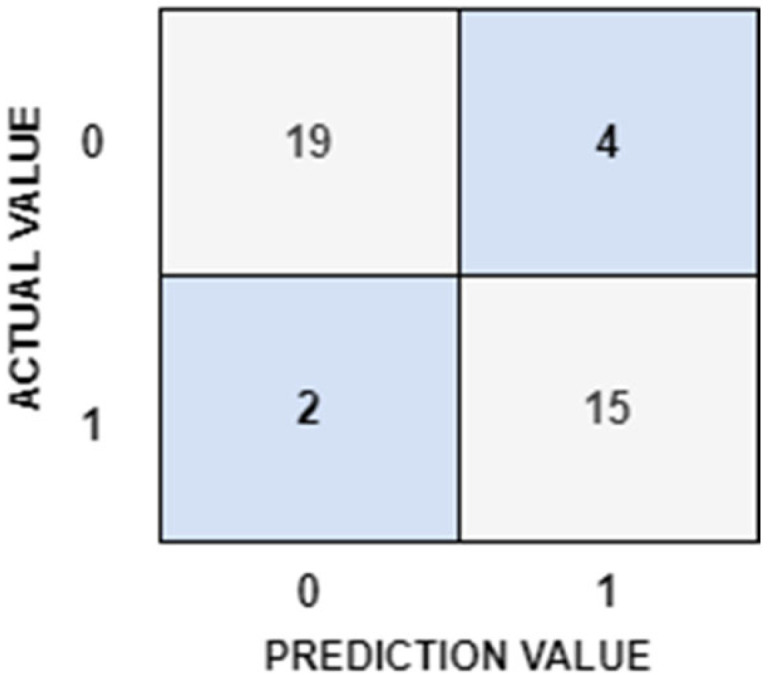
Confusion matrix of VGG19 model with SVM classifier.

Figure 3 illustrates the confusion matrix for the hybrid model, which integrated the VGG19 architecture with the SVM classifier. The high values for true positives and true negatives in the matrix emphasize the model’s effectiveness in distinguishing between the two classes. The overall accuracy was further enhanced by the low numbers of false positives and false negatives. These findings suggest that the proposed hybrid method is a promising tool for clinical applications and provides an efficient solution for the diagnosis of coated tongue.

## 4. Discussion

In this study, we aimed to diagnose coated tongue lesions by applying AI algorithms to intraoral photographs. The results demonstrated that the proposed model exhibited high performance in terms of classification and diagnostic accuracy. This success particularly highlights the potential of AI-based diagnostic systems to bridge the gap between clinical subjectivity and objective assessment—especially in conditions such as coated tongue, which may be overlooked during routine dental examinations. Systemic conditions with oral manifestations, as well as various oral diseases, have been investigated using artificial neural networks trained on intraoral images, and the diagnostic and decision-support capabilities of AI in these contexts have been clearly demonstrated (Table 3) [18,19,20,21,22,23,24].

This table presents a compilation of artificial intelligence-based studies focused on the diagnosis of coated tongue, systematically comparing them in terms of dataset size, classification objectives, applied methods, and reported findings. A common feature among these studies is their methodological focus on identifying coated tongue. Our study proposes a specific diagnostic model aimed at differentiating coated tongue from healthy tongue, achieving high accuracy and F1-score using a VGG-19 + SVM architecture without requiring segmentation. In this respect, it distinguishes itself from other multi-class or region-based analytical studies. Overall, these AI-supported studies on coated tongue detection not only provide promising solutions for clinical decision support systems but also serve as valuable references for the development of future multidimensional diagnostic models.

Kim et al. developed a system for automatic tongue segmentation and coated tongue classification using 711 tongue images obtained through the Digital Tongue Diagnosis System (DTDS). In their study, the tongue region in visual images was segmented using a graph-based segmentation method. The segmented regions were then converted into HSV (hue, saturation, value) color space, and second-order discriminant analysis was applied to classify coating types (white, yellow, mixed, none). When compared with labels provided by traditional medicine practitioners serving as reference standards, the system achieved an accuracy of 85% [18]. In our study, a machine learning classifier (SVM) supported by transfer learning-based deep learning architectures (specifically VGG-19) was developed to distinguish between coated and healthy tongue images. Using a balanced dataset (*n* = 200), the model achieved an accuracy of 85%, sensitivity of 82.6%, specificity of 88.23%, and an F1-score of 86.36%. Both studies demonstrate high accuracy in the automated analysis of tongue images and highlight the clinical potential of digital diagnostic systems. However, our approach offers a more practical and faster solution for clinical settings due to its simplified structure that eliminates the need for segmentation and its hybrid classification strategy.

In the study conducted by Tiryaki et al., a five-class tongue lesion classification was performed, which included 84 specifically labeled coated tongue images. Using a fusion-based majority voting strategy, the developed model achieved an accuracy of 87.36%, a sensitivity of 90.48%, and a specificity of 97.96%. The multi-class structure and increased class diversity introduced additional complexity in distinguishing lesion types [19]. In contrast, our study focused on binary classification, specifically differentiating between coated and healthy tongues. The proposed hybrid model integrated VGG-19-based feature extraction with an SVM classifier and achieved 85% accuracy, 82.6% sensitivity, 88.23% specificity, and an F1-score of 86.36%. While Tiryaki et al.’s model benefited from a larger and more diverse dataset contributing to its overall robustness, our balanced and binary-structured dataset yielded a strong F1 performance by effectively balancing both false positive and false negative rates. Moreover, the high classification performance achieved using a relatively limited dataset and leveraging transfer learning underscores the practicality and potential integration of our model into clinical decision support systems.

Okawa et al. developed a deep learning-based system aimed at automating the traditionally subjective visual assessment of tongue coating in elderly individuals. In their study, tongue region detection was performed using YOLOv2, and the tongue surface was divided into a 7 × 7 grid. Each region was then classified based on its coating score using a ResNet-18-based classification network. The system demonstrated strong agreement with human evaluators (Cohen’s kappa: 0.826, ICC: 0.807), indicating that tongue coating can be reliably assessed in a regional and detailed manner [20]. While the approach by Okawa et al. offers a multidimensional and region-specific analysis, our model provides a faster and more direct diagnostic process. Both methods contribute to clinical decision support at different levels and from complementary perspectives.

Within the framework of the Zheng classification system, which is widely used in Traditional Chinese Medicine (TCM), numerous studies have investigated changes in tongue color, shape, and texture. These studies have employed machine learning techniques to highlight the diagnostic potential of tongue analysis [21,22,23,24]. Zhao et al. applied machine learning methods for tongue segmentation and performed classification of 24 different tongue types using 21 tongue features—including coated tongue—such as color, shape, fissure pattern, and coating thickness. A performance analysis revealed that the Support Vector Machine (SVM) algorithm, optimized with Sequential Minimal Optimization (SMO), achieved the highest accuracy among the tested models [21]. This study underscores the strong classification capability of SVM, particularly in datasets with complex feature representations. While the study by Zhao et al. primarily focused on segmentation and multi-class classification, our approach specifically addresses the binary classification task of distinguishing between coated and healthy tongues. The hybrid architecture integrating deep learning (VGG-19) and machine learning (SVM) in our study enhances diagnostic performance in this specific context. Tang et al. conducted a tongue image classification study within the scope of Traditional Chinese Medicine (TCM), using a dataset of 274 images obtained through the DS01-B system. Of these, 186 images were classified as normal, and 88 as “rot-greasy” tongue coatings according to TCM principles. The classification task involved distinguishing between these two groups, and the study employed a hybrid MI-SVM approach, achieving an accuracy rate of 85% [22]. Similarly, in our study, a comparable accuracy was achieved in differentiating coated and healthy tongues using a hybrid architecture based on VGG-19 and SVM.

In a recent study conducted by Chang et al., efforts were made to objectify tongue diagnosis practices commonly used in TCM. A deep learning-based model was developed to detect various tongue features such as fissures, tooth marks, and thick yellow coatings. The model was trained on a dataset consisting of 764 manually labeled images and implemented using the YOLOv4-tiny object detection algorithm. As a result, a lightweight system capable of real-time operation was obtained, with a model size of only 22.4 MB. The model demonstrated high performance in detecting prominent features such as thick coating and total fissure area, with AP50 scores ranging between 47.67% and 75.92% [23]. While the study by Chang et al. focused on detecting multiple localized features on the tongue surface, our study specifically targeted the binary classification of coated versus healthy tongues. Moreover, in our approach, the deep features extracted from various CNN architectures were analyzed using conventional machine learning algorithms such as SVM, KNN, and MLP for image classification. Although Chang et al.’s study offers significant contributions in terms of visual localization and interpretability of tongue features, our model complements this work by providing a practical classification framework suitable for clinical decision support systems. In the future, a hybrid diagnostic system that combines both approaches could offer not only high diagnostic accuracy but also interpretable features tailored for clinical use.

Li et al. utilized a deep learning framework involving UNet for tongue segmentation and a ResNet-34-based model for classification on a high-resolution dataset comprising 482 tongue images. A total of 11 features—including tongue coating color, thickness, and type—were classified. The system demonstrated strong performance, achieving an overall accuracy of 86.14% and an F1-score of 87.2% [24]. While the study by Li et al. focused on multi-feature classification, our study targeted a more clinically specific issue: the binary classification of coated versus healthy tongue. Despite using a smaller dataset, our carefully designed hybrid architecture, which integrates VGG-19 with SVM, demonstrated strong diagnostic performance, highlighting its potential applicability in clinical practice.

Lo et al. conducted a study aimed at distinguishing patients with stage 0 and stage 1 breast cancer from healthy individuals by utilizing nine tongue features, including tongue color, texture, fissured tongue, coated tongue, red spots, ecchymosis, tooth marks, salivary secretion, and tongue shape. Based on a dataset comprising 137 individuals, the study demonstrated that tongue features may serve as a non-invasive screening tool for the early detection of breast cancer [25]. Similarly, Hsu et al. developed an automated tongue diagnosis system by comparing the tongue features of 199 individuals diagnosed with type 2 diabetes and 372 healthy controls. The same nine features were used in this study, which revealed significant differences in the diabetic group, such as yellow coating, thick coating layers, and bluish tongue color [26]. Furthermore, Deng et al. integrated tongue image features with oral and gut microbiota data to develop a machine learning model for the diagnosis of prediabetes and type 2 diabetes. In that study, tongue imaging parameters such as TB-a and perALL were combined with microbial biomarkers such as Escherichia and Porphyromonas-A. The SVM-based model achieved a diagnostic accuracy of 78.9% and an AUC of 86.9%, indicating strong diagnostic performance [27]. All three studies highlight the value of tongue analysis as a source of robust biomarkers for the diagnosis of systemic diseases and demonstrate the effectiveness of AI-assisted systems in this context. In a similar vein, our study employed an AI-based hybrid architecture for automated tongue analysis, with a specific focus on distinguishing coated tongue from healthy tongue. As coated tongue is often clinically overlooked despite containing potentially valuable information, its accurate identification underscores the potential utility of such technologies not only in the diagnosis of specific lesions but also in broader systemic disease detection.

Jurczyszyn et al. conducted a study using intraoral photographs from 63 participants, including 21 cases of leukoplakia, 21 cases of lichen planus, and 21 healthy volunteers. They applied factor analysis and artificial neural networks (ANNs), reporting a sensitivity of 94% for normal mucosa, 57% for leukoplakia, and 38% for lichen planus. Specificity values were 88% for normal mucosa, 81% for lichen planus, and 74% for leukoplakia [28]. That study provides valuable insight into the applicability of artificial intelligence in distinguishing oral precancerous lesions and aligns with our approach of utilizing intraoral photographs and AI for the diagnosis of coated tongue. Keser et al. evaluated intraoral images of 65 healthy mucosa and 72 oral lichen planus lesions and developed a deep learning approach based on TensorFlow and the Google Inception V3 architecture. Their model distinguished both healthy and diseased mucosa with 100% classification accuracy, strongly highlighting the potential of deep learning in oral diagnostics [29]. This exceptional level of accuracy emphasizes the feasibility of AI-based methods in detecting subtle differences between oral mucosal conditions, even in diagnostically challenging scenarios. Similarly, our proposed algorithm for coated tongue diagnosis also demonstrated high performance in terms of sensitivity, specificity, and overall accuracy. Shamim et al. evaluated the performance of six deep convolutional neural network (DCNN) models using a transfer learning approach for the diagnosis of precancerous tongue lesions and tongue anomalies. Their study successfully classified five specific lesions—including black hairy tongue, fissured tongue, geographic tongue, strawberry tongue, and oral hairy leukoplakia—with high accuracy. The authors concluded that the classification performance of these models was notably strong [30]. In a comparable manner, our study also achieved high accuracy in the diagnosis of coated tongue, demonstrating that AI-based approaches can offer effective solutions for specific clinical challenges.

Wang et al. proposed a deep CNN-based artificial intelligence framework for recognizing tongues with tooth marks. In their study, feature extraction and classification were performed using the ResNet-34 CNN architecture on a dataset of 1548 tongue images, resulting in an impressive accuracy rate exceeding 90% [31]. This study demonstrates the effectiveness of CNN-based approaches in extracting meaningful diagnostic features from tongue images and highlights the growing importance of artificial intelligence in non-invasive medical diagnostics. In our proposed study, a hybrid diagnostic approach was developed by integrating machine learning techniques with a deep neural network structure. VGG-19 was employed for feature extraction, followed by classification using the SVM algorithm, achieving an accuracy of 85%. The lower accuracy compared to the results reported by Wang et al. may be attributed to the differing diagnostic focus of our study, which specifically targeted the distinction between coated and healthy tongue—a more nuanced and potentially challenging classification task. Liang et al. introduced the IF-RCNet model, which was designed to distinguish among thin, normal, and thick tongue types based on a dataset of 1446 tongue images. This two-stage model integrates segmentation and classification tasks using the RCA-UNet for segmentation and RCA-Net for classification, incorporating mixed input and feature fusion strategies. The approach achieved strong results, with an accuracy of 84.87% and an F1-score of 84.22% [32]. While Liang et al.’s study presents a powerful example of morphological analysis based on tongue shape, our model focuses on detecting structural changes in the mucosal surface. In the future, integrating both approaches could contribute to the development of a multidimensional diagnostic system that combines shape-based morphological analysis with mucosa-based lesion classification.

Kusakunniran et al. developed a novel architecture called Deep Upscale U-Net (DU-UNET) for tongue segmentation, which outperformed the conventional U-Net in terms of accuracy and Intersection over Union (IoU). The model was specifically designed for clinical scenarios related to oropharyngeal cancer, where tracking tongue movement is essential. DU-UNET demonstrated superior performance on both publicly available datasets (99.2% accuracy, 97.8% IoU) and their own datasets involving various tongue positions [33]. In contrast, our study adopts a classification-based approach rather than segmentation, aiming to detect the presence of coated tongue through CNN-supported feature extraction and machine learning algorithms. While Kusakunniran et al.’s study focused on precisely delineating tongue boundaries, our work analyzed the mucosal structure of the tongue surface for diagnostic classification purposes. In the future, combining these two approaches could enable the development of a comprehensive, two-stage system that performs both automatic tongue region detection and lesion classification—ultimately offering a more robust solution for clinical diagnostic workflows. More recently, Hosseini et al. proposed a novel convergent metaheuristic algorithm, the Seed Growth Algorithm (SGA), inspired by natural growth processes, to overcome the limitations of gradient-based methods in neural network training [34]. Our proposed CNN-based system could potentially benefit from such advanced optimization strategies to further improve its classification performance in future applications. The integration of metaheuristic approaches like SGA into deep learning-based diagnostic systems represents a promising direction to enhance the robustness and efficiency of these models.

Although it has advantages over the analyzed studies, this study has limitations.

- Despite the promising findings of this study, the relatively small and homogeneous dataset used represents a limitation. Future research should focus on expanding the dataset to include diverse populations and systemic conditions, which would further enhance the robustness and generalizability of the proposed model.
- Deep learning models were used to extract features. No end-to-end retraining (fine-tuning) was performed. This may limit the scope of the model. In future studies, full model training can be tried together with transfer learning.
- In this study, the models were only examined using accuracy, sensitivity, and other statistical measures. However, how the models perform in real clinical conditions has not yet been tested. This can be tested in a clinical setting in future studies.

## 5. Conclusions

Regular tongue examinations are essential for monitoring both oral and general health, playing a strategic role in the early detection and management of potential health issues. Recognizing benign lesions that do not require treatment or further evaluation helps avoid unnecessary tests and ensures more efficient patient care. This study highlights the importance of leveraging AI-driven systems to address clinical challenges, particularly for diagnosing overlooked and asymptomatic conditions like coated tongue, which are often neglected during routine dental evaluations. Rapidly advancing AI technologies offer transformative opportunities in recognizing asymptomatic and difficult-to-diagnose lesions, developing tailored treatment strategies, and enhancing patients’ quality of life. This study presents a novel and practical solution for diagnosing coated tongue lesions using AI-based methodologies. With further validation and larger-scale studies, such systems could play a pivotal role in improving oral diagnostics and potentially contribute to the early detection of systemic diseases and malign conditions.

## Figures and Tables

**Table 1:** Performance evaluation metrics.

Metrics	Formulas
**Accuracy (Acc)**	(TP + TN)/(TP + TN + FN + FP)
**Sensitivity (Sen)**	TP/(TP + FN)
**Specificity (Spe)**	TN/(TN + FP)
**F1 Score**	2TP/(2TP + FN + FP)

**Table 2:** Classification results.

Classifier	Model	Sen (%)	Spe (%)	Acc (%)	F1 Score (%)
**SVM**	VGG16	86.36	83.33	85	86.36
	**VGG19**	**82.6**	**88.23**	**85**	**86.36**
	ResNet	61.11	81.81	72.5	66.66
	MobileNet	83.33	86.36	85	83.33
	NasNet	66.66	95.45	82.5	77.41
**KNN**	VGG16	77.77	77.27	77.5	75.67
	VGG19	77.77	77.27	77.5	75.67
	ResNet	50	86.36	70	60
	MobileNet	73.07	78.57	75	79.16
	NasNet	73.91	94.11	82.5	82.92
**MLP**	VGG16	100	50	77.5	83.01
	VGG19	82.6	88.23	85	86.36
	ResNet	68.18	66.66	67.5	69.76
	MobileNet	83.33	72.72	77.5	76.92
	NasNet	77.27	88.88	82.5	82.92

**Table 3:** Comparison of AI-Based Studies on Coated Tongue Imaging.

Authors	Dataset	Objective	Methods	Results
Tiryaki et al. [19]	A total of 623 tongue images, including 84 coated tongue images	Classification of various tongue lesions, including coated tongue, fissured tongue, and others.	Deep learning model with majority voting	For coated tongue: an accuracy of 87.36%, sensitivity of 90.48%, and specificity of 97.96%
Tang et al. [22]	274 samples of tongue images, 186 normal tongue coating images and 88 rotten-greasy ones	Tongue coating classification based on Traditional Chinese Medicine (TCM)	MI-SVM	An accuracy of 85% was achieved in differentiating between coated and normal tongues
Okawa et al. [20]	395 tongue images	Segmental analysis of tongue coating	YOLOv2 + ResNet-18	High level of agreement with human evaluators, with a kappa coefficient of 0.826
Zhao et al. [21]	Interpretative literature review; the sample size was not specified.	Classification of tongue characteristics based on Traditional Chinese Medicine (TCM), including color, fissures, shape, coating thickness, and type, etc.	SVM optimized using the Sequential Minimal Optimization (SMO) algorithm	A total of 24 features, including coated tongue, were successfully classified with high accuracy
Li et al. [24]	482 tongue images	Classification of tongue characteristics based on Traditional Chinese Medicine (TCM), including color, fissures, shape, coating thickness, and type, etc.	UNet + ResNet-34	An accuracy of 86.14% was achieved in the classification of tongue coating type, thickness, and color
Kim et al. [18]	711 tongue images	Tongue segmentation and coated tongue classification	Graph-based segmentation combined with HSV color space and discriminant analysis	An accuracy of 85% was achieved for the presence and type of tongue coating
Chang et al. [23]	696 images with thick and yellow tongue coating/764 total labeled tongue images	Classification of tongue characteristics based on Traditional Chinese Medicine (TCM), including color, fissures, shape, coating thickness, and type, etc.	YOLOv4-tiny	AP50: 75.92% for thick coating, 47.67% for yellow coating; real-time detection model
This study	A total of 200 images, including 100 with coated tongue and 100 from healthy subjects	Coated tongue diagnosis and differentiation between healthy and coated tongue	VGG-19 + SVM	An accuracy of 85% and an F1-score of 86.36% were achieved.

## Data Availability

The data supporting the findings of this study are available from the corresponding author (S.C.B.) upon request. The data were not publicly available because they contained information that could compromise the privacy of the participants.
